# Systematic Identification of Cellular Signals Reactivating Kaposi Sarcoma–Associated Herpesvirus

**DOI:** 10.1371/journal.ppat.0030044

**Published:** 2007-03-30

**Authors:** Fuqu Yu, Josephine N Harada, Helen J Brown, Hongyu Deng, Moon Jung Song, Ting-Ting Wu, Juran Kato-Stankiewicz, Christian G Nelson, Jeffrey Vieira, Fuyuhiko Tamanoi, Sumit K Chanda, Ren Sun

**Affiliations:** 1 Department of Molecular and Medical Pharmacology, University of California Los Angeles, Los Angeles, California, United States of America; 2 Genomics Institute of the Novartis Research Foundation, San Diego, California, United States of America; 3 Department of Microbiology, Immunology and Molecular Genetics, University of California Los Angeles, Los Angeles, California, United States of America; 4 School of Dentistry, University of California Los Angeles, Los Angeles, California, United States of America; 7 Department of Laboratory Medicine, University of Washington, Seattle, Washington, United States of America; Washington University School of Medicine, United States of America; 5 Institute of Biophysics, Chinese Academy of Sciences, Beijing, China; 6 Division of Biotechnology, College of Life Sciences and Biotechnology, Korea University, Seoul, Republic of Korea

## Abstract

The herpesvirus life cycle has two distinct phases: latency and lytic replication. The balance between these two phases is critical for viral pathogenesis. It is believed that cellular signals regulate the switch from latency to lytic replication. To systematically evaluate the cellular signals regulating this reactivation process in Kaposi sarcoma–associated herpesvirus, the effects of 26,000 full-length cDNA expression constructs on viral reactivation were individually assessed in primary effusion lymphoma–derived cells that harbor the latent virus. A group of diverse cellular signaling proteins were identified and validated in their effect of inducing viral lytic gene expression from the latent viral genome. The results suggest that multiple cellular signaling pathways can reactivate the virus in a genetically homogeneous cell population. Further analysis revealed that the Raf/MEK/ERK/Ets-1 pathway mediates Ras-induced reactivation. The same pathway also mediates spontaneous reactivation, which sets the first example to our knowledge of a specific cellular pathway being studied in the spontaneous reactivation process. Our study provides a functional genomic approach to systematically identify the cellular signals regulating the herpesvirus life cycle, thus facilitating better understanding of a fundamental issue in virology and identifying novel therapeutic targets.

## Author Summary

Kaposi sarcoma is a cancer that commonly occurs in AIDS patients. The tumor-associated virus, Kaposi sarcoma–associated herpesvirus, has two distinct phases in its life cycle: inactive latency and active lytic replication. The balance between these two phases is critical for viral pathogenesis. Cellular signals play a role in the switch from latency to lytic replication, termed reactivation. To systematically evaluate the cellular signals regulating this reactivation process in Kaposi sarcoma–associated herpesvirus, a genome-wide cDNA library screen was conducted. Twenty-six thousand mammalian genes were individually expressed in cells that harbor the latent virus, and their effect on reactivation was assessed through a sensitive reporter system. A group of diverse cellular signaling proteins were identified and validated. Further analysis revealed that the activation of the cellular Raf/MEK/ERK/Ets-1 pathway is shared by multiple upstream inducers to trigger reactivation. This work provides a functional genomic approach to systematically identify the cellular signals regulating the herpesvirus life cycle, thus facilitating better understanding of a fundamental issue in virology and identifying novel therapeutic targets.

## Introduction

Kaposi sarcoma–associated herpesvirus (KSHV), also known as human herpesvirus-8 (HHV-8), is a member of the gamma-herpesvirus family. This virus family also includes the Epstein–Barr virus (EBV) and murine gamma-herpesvirus 68 [1–4]. Herpesviruses have two distinct phases in their life cycle: latency and lytic replication. During latency, the viral genome is replicated by cellular DNA polymerase, and only a few gene products are expressed. One of the advantages of latency is the ability of the virus to evade the host immune responses. After stimulation, the virus can enter the lytic cycle by a reactivation process. Genes that are induced in the lytic phase can be classified as immediate-early genes, early genes, and late genes according to their temporal expression pattern and sensitivity to viral protein synthesis and DNA replication inhibitors. Upon replication of the viral genome by a viral DNA polymerase, viral progeny are produced, frequently resulting in cell death.

The distinctive features of gamma-herpesviruses include their ability to establish long-term infections in lymphocytes, and their oncogenic potential. EBV is associated with nasopharyngeal carcinoma, Burkitt lymphoma, Hodgkin disease, and other types of malignancies [5,6]. KSHV is associated with Kaposi sarcoma, primary effusion lymphoma (PEL), and some forms of multicentric Castleman disease [2,7–11]. Viral infection persists predominantly in a latent form in tumor cells. However, lytic replication is believed to play a critical role in tumorigenesis. It is likely that continuous low-level reactivation leads to efficient viral transmission and spread, and subsequently disease development in a subset of the infected cells. Cytokines of both viral and cellular origin produced during lytic replication may provide a favorable environment for the proliferation of infected cells [12–16].

The switch between latency and lytic replication has been actively investigated. KSHV replication and transcription activator (RTA), a protein product encoded mainly by open reading frame (ORF) 50, plays a central role in regulating this switch in KSHV [17–22]. In latently infected cells, the expression of RTA is necessary and sufficient to disrupt KSHV latency and trigger the complete lytic replication process. RTA functions as a transcription factor, activating expression of multiple downstream target genes as well as its own gene [23,24]. Among these downstream effector genes is the early viral transcript polyadenylated nuclear RNA (PAN, also called nut-1). PAN is the most abundant transcript made during the lytic cycle, and is directly induced by RTA [25–28].

RTA contains an N-terminal DNA-binding domain and a C-terminal activation domain. The N-terminal DNA-binding domain mediates sequence-specific DNA binding. RTA response elements (RREs) have been identified within several lytic gene promoters, including the PAN, v-IL-6, ORF57, and Kpsn promoters [16,29–31]. The RRE within the PAN promoter was incorporated into a highly sensitive luciferase reporter construct named pPAN-69Luc. RTA has also been shown to interact with several transcription modulatory proteins to maximally facilitate lytic gene expression, including CREB-binding protein (CBP), the SWI/SNF chromatin remodeling complex and the TRAP/Mediator coactivator, and CSL, a target of the Notch signaling pathway [32–35]. RTA functionally interacts with other viral proteins as well [36].

Although the function of RTA in KSHV reactivation has been extensively studied, the cellular pathways involved in regulating transcription and expression of RTA have not been systematically studied. In an attempt to systematically identify these signals, we carried out a genome-wide cell-based screen utilizing an arrayed cDNA expression library. The screen was conducted in KS-1 cells (a PEL cell line latently infected with KSHV, a twin cell line of BC-3 cell line) in a 384-well format. The pPAN-69Luc reporter construct is highly responsive to RTA and was therefore used as an indicator of KSHV reactivation. The screen assessed the effect of ectopic expression of 26,000 individual cDNA clones on RTA-dependent reporter activity and identified a list of positive cellular genes. We then conducted more extensive analyses on one of the most potent reactivators, Ras, and investigated the signaling components downstream of Ras to elucidate the underlying molecular mechanisms for reactivation.

## Results

### A Large-Scale Screen with a Reporter System

**Figure 1:**
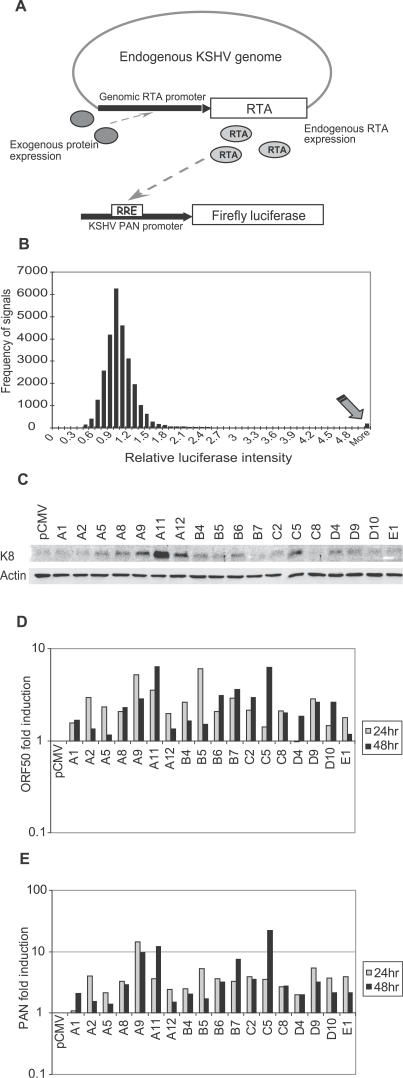
Schematic Representation and Results of the Primary Screen System (A) Overview of the screen approach. Individual cDNA clones were transfected into KSHV latently infected KS-1 cells in 384-well plates. Positive signals activate the RTA promoter and induce the expression of RTA protein from the endogenous viral genome. RTA can also activate its own promoter. In response to the endogenous RTA expression, the PAN promoter in the cotransfected PAN-69Luc reporter construct will be activated and the reporter activity can be analyzed. (B) Histogram of a representative screen result. The *x*-axis represents the relative luciferase intensity generated by transfected cDNAs normalized to the median luciferase intensity; the *y*-axis represents the frequency of signals falling in the binned range of luciferase intensity. The arrow indicates the group of signals that showed greater than a 5-fold increase in luciferase intensity. (C–E) Western blot showing the induction of KSHV lytic protein K8 expression; RT-Q-PCR showing the induction of lytic transcripts RTA/ORF50 (D) and PAN (E) upon transfection of the cDNA clones in the top hits list into KS-1 cells.

To systematically identify the cellular signals that induce RTA activity (and therefore reactivate KSHV), a reporter system was established (Figure 1A). pPAN-69Luc has low basal activity in the absence of RTA and is highly sensitive to RTA in a dose-dependent manner (Figure S1) [25]. The screen design involved cotransfection of the 26,000 mammalian cDNA clones individually into KS-1 cells with pPAN-69Luc. A very large amount of cDNA was used in the high-throughput screen to increase the sensitivity. Activation of the endogenous RTA promoter on the viral genome by exogenous cDNA gene products would cause a chain of events culminating in luciferase expression from the RTA-dependent pPAN-69Luc reporter.

The screen results are summarized in Figure 1B. The majority of the reporter signals cluster within a narrow range between 0.5- to 2-fold of the median luciferase activity, whereas a small number of cDNAs induced luciferase expression activity greater than 5-fold over the median (indicated by the arrow). There were only a very limited number of signals detected between these two groups, indicating the significance of the outliers. The signals in the outlier group were considered as positive signals.

To validate the ability of these cDNA gene products to induce reactivation, we transfected each cDNA individually into KS-1 cells and assessed KSHV lytic gene expression levels by Western blot and reverse transcription–quantitative PCR (RT-Q-PCR) analysis. Lower ratios of DNA to cells were used in the verification experiments (Figure 1C–1E; Table 1). The verified list contains a number of signaling molecules, either kinases or transcription factors. Among them was a previously identified molecule that reactivates KSHV, the catalytic subunit of protein kinase A (PKA, “A9”), which was one of the molecules that gave the strongest signal in the screen [37]. Another robust inducer, Kirsten rat sarcoma 2 viral oncogene homolog (v-Ki-ras2, “A11”), is an important upstream signaling molecule that regulates cellular functions via a number of distinct pathways. In addition to PKA and Ras, some other positive cellular cDNA clones are: 1) XBP1: encodes X-box binding protein 1, a B cell differentiation factor that is involved in late stage of B cell terminal differentiation and the unfolded protein response [38,39]; 2) Zfp64: encodes mouse zinc finger protein 64, a nuclear protein which has been reported to be involved in transcription regulation [40]; 3) NR4A1: encodes a member of the steroid-thyroid hormone-retinoid receptor superfamily and acts as a nuclear transcription factor. It has been reported to be related to TPA (12-O-tetradecanoylphorbol-13-acetate) and VP-16–induced apoptosis [41]; 4) Pitx1: encodes a mouse paired-like homeodomain transcription factor 1, which is found to be a cofactor of AP-1 [42,43]; 5) Nfib: encodes mouse nuclear factor I/B, an important transcription factor in development [44]; 6) Rit1: encodes a Ras-like protein expressed in many tissues [45]; 7) mouse Ets-1 and Etv1 (mouse Ets variant gene 1). The Ets family proteins are among the transcription factors downstream of Ras and are involved in various biological functions, including regulation of cell proliferation, angiogenesis, and immune response [46,47]; and 8) Gadd45a: encodes mouse growth arrest and DNA-damage-inducible 45 alpha. GADD45 proteins are induced by ultraviolet radiation (UV), irradiation, stress-related pathways, histone deacetylase (HDAC), and prostaglandin J2 [48,49]. Some of these cellular proteins are functionally connected to known physical and chemical herpesvirus reactivation inducers such as UV, stress, and TPA.

**Table 1:**
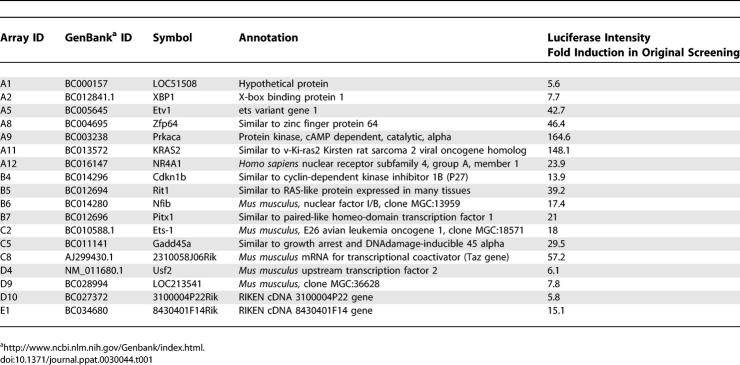
Cellular Genes That Activated KSHV

Western blot analysis showed that at the 48-h time point, the majority of the cDNA clones induced lytic protein K8 expression (Figure 1C). The RT-Q-PCR analysis at 24 h or 48 h post-transfection also showed that the majority of the cDNA clones induced the transcript levels of immediate-early and early genes RTA/ORF50 and PAN, strongly suggesting that they are able to reactivate KSHV. Our results suggest that the reporter screen system accurately identified cellular gene products able to reactivate KSHV. Further studies on the underlying mechanisms of these genes would reveal large amount of information on regulation of KSHV reactivation by the cellular signaling network.

### The Raf/MEK/ERK Pathway Mediates Ras-Induced KSHV Reactivation through Activating the RTA Promoter

As one of the strongest inducers in our screen system and an important signaling molecule, v-Ki-ras2 was selected for follow-up analysis in this study to further verify the systematic approach and examine the molecular mechanisms underlying the strong reactivation effect. v-Ki-ras2 is a member of the *ras* gene family, which consists of three members: Ha-ras, Ki-ras, and N-ras. These genes encode highly related small GTP-binding proteins, which are important upstream signaling components of several cellular pathways involved in cell proliferation, differentiation, stress responses, and apoptosis. All Ras proteins have an identical Ras effector binding domain, which is responsible for interacting with all known downstream signaling molecules, including Raf and PI3K [50]. To verify and characterize the effect of Ras on KSHV reactivation, we utilized two different constitutively active forms of Ras: v-Ki-ras2 and Ha-ras (Q61L).

**Figure 2:**
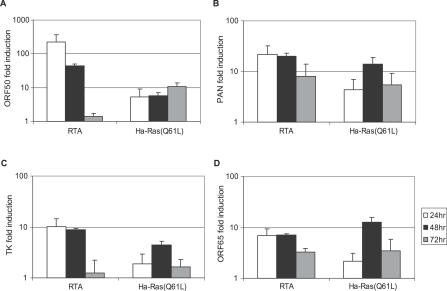
Effect of Ras on KSHV Lytic Transcript Levels Assessed by RT-Q-PCR BC-3 cells were transfected with pCMV, RTA, or Ha-Ras (Q61L). At 24, 48, and 72 h post-transfection, levels of immediate-early lytic transcripts RTA/ORF50 (A), early lytic transcripts PAN (B), viral thymidine kinase (TK) (C), and late lytic transcript ORF65 (D) were measured by RT-Q-PCR. (The high level of ORF50 transcript level in the RTA transfected cells in [A] is largely due to the transfection of exogenous RTA cDNA expression clone.)

**Figure 3:**
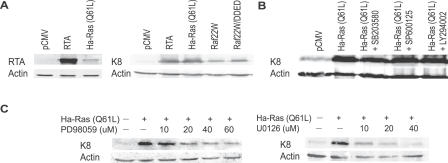
Effect of Chemical Inhibitors on KSHV Reactivation in PEL Cells (A) Expression of KSHV lytic protein RTA or K8 24 h after transfection with pCMV, RTA, Ha-Ras (Q61L), Raf22W, and Raf22W/DDED. (B) The effects of the p38 pathway inhibitor SB203580 (20 uM), the JNK pathway inhibitor SP600125 (20 uM), and the PI3K/Akt pathway inhibitor LY294002 (20 uM) on Ras-induced K8 expression. (C) Dose-dependent inhibition of lytic protein K8 expression upon treatment with the Raf/MEK/ERK pathway–specific inhibitors PD98059 and U0126.

An RT-Q-PCR analysis was conducted to determine if lytic transcript levels increased when Ras was ectopically expressed (Figure 2). The results demonstrated that Ha-Ras (Q61L) can induce KSHV immediate early (RTA/ORF50) to more than 10-fold. The RT-Q-PCR data also showed the induction of early (PAN and viral thymidine kinase) and late (ORF65) lytic transcripts 5- to over 10-fold. The induction of ORF65 indicated the onset of lytic viral DNA replication, which is consistent with the previous finding that induction of RTA is necessary and sufficient to trigger the complete lytic replication process [20,28]. The activation of the KSHV lytic program by Ras was also confirmed by Western blot and immunofluorescence assay (Figures 3A and S2).

The signals transduced by Ras diverge into multiple pathways, including mitogen-activated kinase (MAPK) pathways and the PI3K/Akt pathway. To explore which pathways are directly involved in transducing the reactivation signal, different MAPK and PI3K inhibitors were applied to cells transiently transfected with Ras. Western blot analysis revealed that the early lytic protein K8 expression induced by the ectopic expression of Ha-Ras (Q61L) was not affected by p38 inhibitor SB203580, JNK inhibitor SP600125, or PI3K/Akt inhibitor LY294002 (Figures 3B and S4). In contrast, when an increasing amount of the inhibitors specific to the Raf/mitogen-activated protein (MAP)/extracellular signal–regulated kinase (ERK) kinase (MEK)/ERK (Raf/MEK/ERK) pathway (PD98059 and U0126) was applied, the induction of K8 expression by Ras was inhibited in a dose-dependent manner (Figure 3C). Moreover, constitutively active mutants of Raf (Raf22W and Raf22W/DDED) [51]induced K8 expression (Figure 3A). Though it is difficult to compare the robustness of induction by Ras and Raf expression due to different expression levels and induction kinetics of different cDNA constructs, our results clearly showed that the Ras/Raf/MEK/ERK pathway is the main pathway through which Ras exerts its effect on KSHV reactivation.

**Figure 4:**
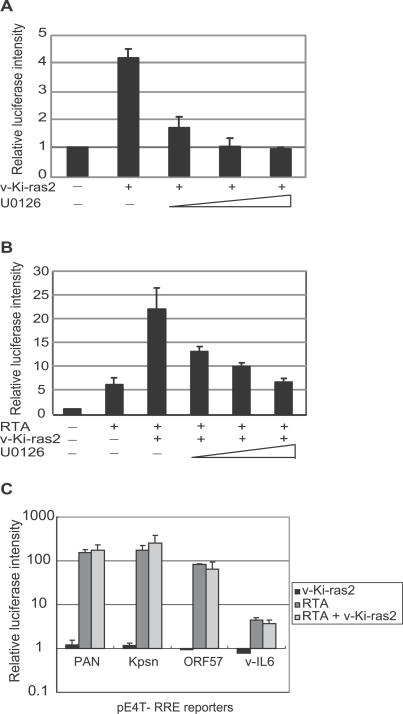
Effect of Ras on KSHV Lytic Promoter Activity Assessed by Reporter Assays (A and B) The effect of increasing doses of U0126 (5 uM, 10 uM, 20uM) on Ras-induced RTA promoter activity in the absence (A) or presence (B) of endogenous RTA protein. (C) The effect of Ras on the promoter activity of four KSHV lytic genes PAN, Kpsn, ORF57, and v-IL-6 in the absence or presence of RTA protein. The fold induction represents the increase in luciferase intensity corrected over the background induction on pE4T basic construct. The data presented here are the average of three independent experiments. The promoter activity is the relative luciferase intensity compared to pcDNA3-transfected cells whose luciferase intensity was set as 1.

To explore the mechanism by which the Ras/Raf/MEK/ERK pathway reactivates KSHV, reporter assays were conducted on the RTA promoter in 293T cells. The results showed that v-Ki-Ras2 activated the RTA promoter, and this effect was inhibited by U0126 in a dose-dependent manner (Figure 4A). It has also been shown that the RTA protein can activate its own promoter [23]. In 293T cells where there is no endogenous RTA protein, the cotransfection of v-Ki-ras2 with RTA resulted in an enhanced activation of the RTA promoter. This effect was inhibited by U0126 to a level similar to that induced by the RTA protein alone (Figure 4B), suggesting that RTA and v-Ki-Ras2 activate expression from the RTA promoter by two distinct mechanisms. To examine the effect of Ras on other downstream lytic viral promoter activation, reporter assays were conducted in 293T cells using the reporter constructs pE4T-PAN, pE4T-Kpsn, pE4T-ORF57, and pE4T-v-IL-6, which contain the RRE sequences in the respective lytic promoters. The results showed that RTA significantly induced their luciferase activity [16,26,29–31]. However, v-Ki-Ras2 did not have significant effect on any of these promoters, either by its own expression or by co-expression with RTA (Figure 4C). Collectively, our reporter assays suggested that the Ras/Raf/MEK/ERK pathway can activate the RTA promoter, which further leads to the activation of downstream lytic genes. The delayed induction of the levels of viral transcripts by Ha-Ras (Q61L) compared to RTA was also consistent with this hypothesis (Figure 2). We have further determined that the DNA sequences mediating the Ras and RTA responses are located in different regions on the RTA promoter (unpublished data).

### The Raf/MEK/ERK Pathway Mediates TPA-Induced KSHV Reactivation and Spontaneous Reactivation in PEL Cells

**Figure 5:**
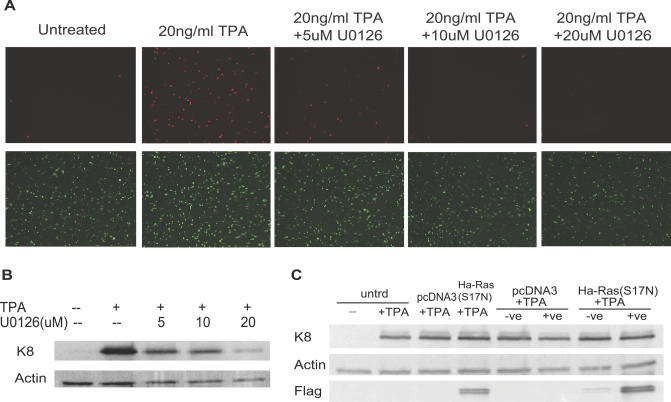
Effect of TPA on KSHV Reactivation in PEL Cells (A) Fluorescence microscopy of rKSHV.219 latently infected JSC-1 cells after 48 h incubation with TPA (20 ng/ml) and increasing amount of U0126 pretreatment. (B) The expression of KSHV lytic protein K8 after 24 h incubation with TPA (20 ng/ml) and increasing amount of U0126 pretreatment. (C) BC-3 cells were cotransfected with MACS4.1 plasmid Ha-Ras (S17N) (Flag-tagged) 24 h before incubation with TPA (20 ng/ml). Sixteen hours later, the successfully transfected cells were then enriched by a MACSelection system as +ve portion, and untransfected cells were indicated as −ve portion. Ha-Ras (S17N) and K8 expression was assessed by Western blot analysis with anti-Flag and anti-K8 antibody, respectively.

The phorbol ester TPA is a commonly used chemical inducer of herpesvirus reactivation [52]. We examined the mechanism of TPA-induced KSHV reactivation in JSC-1 cells that are latently infected with a KSHV reporter virus, rKSHV.219. This recombinant virus expresses the red fluorescent protein from the KSHV lytic PAN promoter as an indicator of reactivation, and the green fluorescent protein (GFP) from the EF-1alpha promoter as an internal control [53]. TPA treatment activated the transcription of the lytic gene PAN, and this induction was blocked by U0126 pretreatment in a dose-dependent manner (Figure 5A). Western blot of KS-1/BC-3 cells also showed that the TPA-induced expression of K8 was blocked by U0126 treatment, consistent with previous RT-PCR results (Figure 5B) [54]. In contrast, reactivation induced by another commonly used chemical, sodium butyrate, was not affected by U0126 (Figure S3A), suggesting that the role of the Raf/MEK/ERK pathway is specific to the effect of TPA. Consistent with our hypothesis that the Raf/MEK/ERK pathway reactivates virus in an RTA-dependent manner, an RTA-null virus failed to express lytic genes and produce infectious viral particles upon TPA treatment [55]. The Raf/MEK/ERK pathway can be activated by multiple upstream signals [56]. We demonstrated that Ha-Ras (S17N), a dominant-negative mutant of Ras, was not able to inhibit TPA-induced K8 expression, which suggests that TPA induces KSHV reactivation by a Ras-independent mechanism (Figure 5C). By examining the mechanisms of Ras- and TPA-induced KSHV reactivation, we revealed that different upstream signals can converge into one pathway to mediate herpesvirus reactivation.

**Figure 6:**
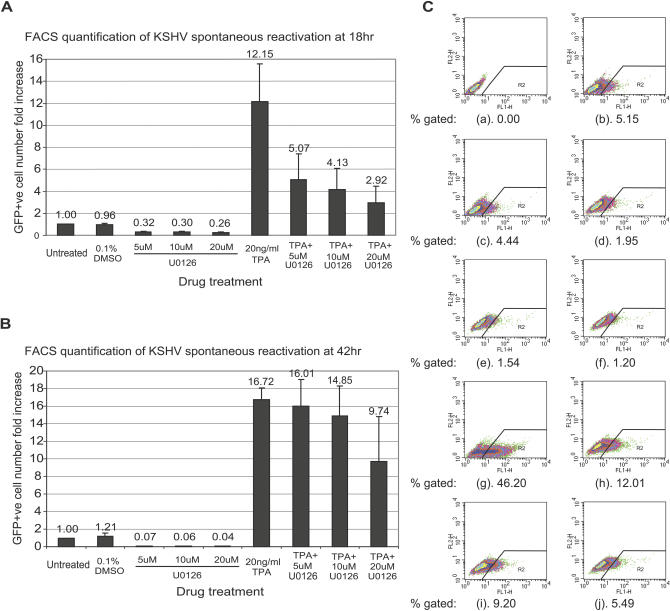
Effect of U0126 on KSHV Spontaneous Reactivation in BC-3-G Cells by Flow Cytometry Three independent experiments were conducted at 18 h (A) and 42 h (B) time points; a representative experiment result for the 18-h time point is shown here (C): (a) untreated parental cell line BC-3; (b) BC-3-G untreated; (c) BC-3-G + 0.1% dimethyl sulfoxide (DMSO); (d) BC-3-G + 5 uM U0126; (e) BC-3-G + 10 uM U0126; (f) BC-3-G + 20 uM U0126; (g). BC-3-G + 20 ng/ml TPA; (h) BC-3-G + 20 ng/ml TPA + 5 uM U0126; (i) BC-3-G + 20 ng/ml TPA + 10 uM U0126; (j) BC-3-G + 20 ng/ml TPA + 20 uM U0126.

We further investigated whether the Raf/MEK/ERK pathway also contributes to KSHV spontaneous reactivation. Although KSHV persists predominantly in the latent form in Kaposi sarcoma tumors and PEL, low frequency spontaneous reactivation can be detected in both Kaposi sarcoma tumor cells [10,57,58]and B cells [10,28,59–62]. The cause of spontaneous reactivation is not yet clear for any herpesvirus, but it is believed to be important for virus-associated disease pathology and transmission. To more sensitively measure spontaneous reactivation rate, we established a BC-3-based reporter cell line, BC-3-G, with GFP expression driven by the PAN promoter. The PAN promoter is very sensitive and specific to the presence of viral RTA protein, but is not activated at all by other cellular factors, as indicated by a lack of regulation by any of the 26,000 cDNA clones in the genome-wide cDNA screen in the absence of viral RTA activation. Thus, the activation of the PAN promoter in BC-3-G cells serves as a specific indicator of KSHV spontaneous reactivation. KSHV spontaneous reactivation in these cells was assessed by flow cytometry (Figure 6). We found that upon U0126 treatment, the percentage of GFP-positive cells (gated in the R2 region) was significantly reduced. The total cell population had a clearly “shrunken tail” of GFP expression. The percentage of GFP-positive cells in the total cell population is consistent with the spontaneous and induced reactivation rate that was previously reported in PEL cells [62]. The ratio of inhibition by U0126 remained similar even if we set the fluorescence-activated cell sorting (FACS) gate value up or down 2-fold. This result indicated that the Raf/MEK/ERK pathway is an important factor regulating KSHV spontaneous reactivation in lymphoma cells. To our knowledge, our result is the first example that has revealed a specific cellular signaling pathway regulating spontaneous reactivation of a herpesvirus.

### Ets-1 Mediates Ras- and TPA-Induced Reactivation

**Figure 7:**
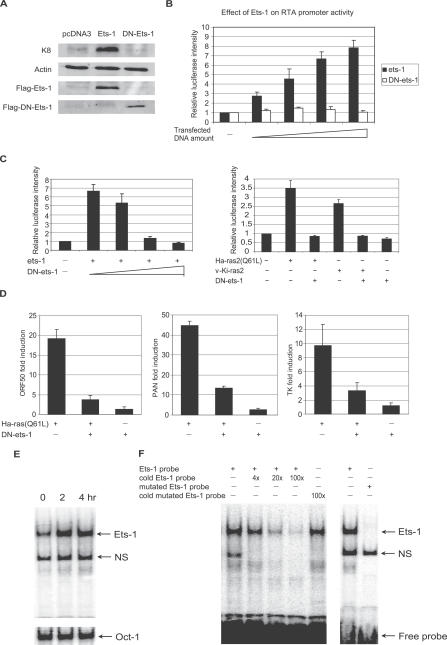
Effect of Ets-1 on KSHV Reactivation by Western Blot, Reporter Assays, and RT-Q-PCR (A) Expression of K8 protein in BC-3 cells 48 h after transfection of ets-1 or DN-ets-1. Expression of Ets-1 and DN-Ets-1 was detected using an anti-Flag antibody. (B) The activation of the RTA promoter upon expression of increasing amount of Ets-1 or DN-Ets-1 (10 ng, 50 ng, 100 ng, and 250 ng) in 293T cells by reporter assay. (C) The effect of increasing amount of DN-Ets-1 on RTA promoter activity induced by Ets-1 (left), Ha-Ras (Q61L), or v-Ki-Ras2 (right). The firefly luciferase readings were normalized to Renilla luciferase reading, and the relative luciferase induction compared to vector alone was presented here. (D) Levels of lytic transcripts RTA/ORF50, PAN, and viral thymidine kinase (TK) upon transfection of Ha-Ras (Q61), DN-Ets-1, or a combination of both. (E) EMSA assessing Ets-1 DNA binding ability using BC-3 cell nuclear extract after incubation with TPA (20 ng/ml) for indicated time. The binding of Oct-1 probe was used as loading control. NS, non-specific binding. (F) Competition experiments confirming the specificity of binding to Ets-1 probe. NS, non-specific binding.

Ets family transcription factors are potential downstream effectors of Raf/MEK/ERK pathway. There are two major groups of proteins in the Ets family: the Ets group, which includes Ets-1, Ets-2, and Pointed; and the ternary complex factors (TCFs), which include Elk1, Sap1a, Sap1b, Fli1, and Net (reviewed in [63]). The identification of mouse Ets-1 and Etv1 from the cDNA screen led us to examine the effect of Ets-1 protein in KSHV reactivation. We found that K8 expression was induced by ectopic expression of Ets-1 in KS-1/BC-3 lymphoma cells, but not by a dominant negative Ets-1 mutant (DN-Ets-1), which contains the DNA-binding domain but lacks the transactivation domain (Figure 7A) [64]. Ets-1, but not DN-Ets-1, was also able to activate the RTA promoter (Figure 7B). Furthermore, DN-Ets-1 inhibited Ras-induced RTA promoter activation and KSHV lytic transcript levels (Figure 7B–7D). Western blot analysis also showed that DN-Ets-1 expression reduced TPA-induced K8 expression (Figure S3B), suggesting that both Ras- and TPA-initiated KSHV reactivation is mainly mediated by Ets-1.

To examine whether activation of the Raf/MEK/ERK pathway leads to endogenous Ets-1 activation in PEL cells, electrophoretic mobility shift assay (EMSA) was conducted with nuclear extracts from BC-3 cells. The results showed that Ets-1 DNA binding ability was increased more than 2-fold as early as 2 h after TPA treatment. It was a significant increase, considering that viral lytic replication was only induced in a subset of the cell population. In comparison, the Oct-1 binding activity did not change at all (Figure 7E). The specificity of the Ets-1 binding was verified by competition experiments with specific and mutant probes and cold oligonucleotides (Figure 7F). The data provided additional evidence for the involvement of Ets-1 in reactivation induced by Raf/MEK/ERK pathway activation.

## Discussion

It is generally hypothesized that multiple cellular signaling pathways regulate herpesvirus reactivation in latently infected cells. In this study, we address this issue directly, utilizing a genome-scale analysis to identify cellular factors that can reactivate endogenous herpesvirus in latently infected B cells. By using this approach, we were able to identify diverse signaling molecules as activators of KSHV reactivation. The effectiveness of this genome-wide analysis was validated by revealing the critical role of the Ras/Raf/MEK/ERK/Ets-1 pathway in mediating signals from the cell surface to the viral genome in the nucleus to reactivate KSHV. This pathway is also crucial for TPA-induced reactivation and spontaneous reactivation.

Our primary screen was designed to measure the activation of the endogenous RTA promoter, using a reporter generated from a downstream transcriptional target of RTA, PAN. The PAN promoter provides a robust measure for RTA function, and further enabled us to capitalize on three rounds of signal amplification in our screen output: the direct activation of the RTA promoter by the transfected gene product, autoactivation of the RTA promoter by RTA itself, and subsequent transactivation of the PAN promoter by RTA. This substantially increased the sensitivity of our approach, and enabled us to overcome typically low transfection efficiencies observed in the lipid-mediated transfection of lymphoid cells. At the same time, the PAN reporter construct pPAN-69Luc contains only a minimum promoter region (69 base pair) that is very sensitive to RTA expression. This short sequence makes it less likely for other transcription factors to bind to and activate the PAN promoter. Indeed, the cDNA clones identified in our screen did not activate PAN promoter in 293T cells in the absence of the viral genome, as shown for v-Ki-Ras2 in Figure 4C. Our screen identified a wide range of cellular gene products that were able to increase RTA expression and thereby initiate viral reactivation. Defining the consequences of multiple inputs is often too difficult to address using traditional methodologies, but has become a new exciting topic in systems biology. The multiple signals that have been identified in the high-throughput screen, in combination with our sensitive fluorescent/luminescent reporter cell systems, can be utilized to further characterize their combinatory effect. This also forms a unique platform, to which other highly efficient interdisciplinary approaches, such as microfluidic optical and algorithm-based control systems, can be applied to explore viral processes at single cell and population levels.

In latently infected cells, virus reactivation is thought to be the consequence of a disruption of the “balance” between signaling pathways that induce or suppress virus lytic replication. Our screen system utilizes an ectopic expression strategy to achieve the disruption of the balance of the signaling network, thus exposing the virus to certain pathways that are involved in virus reactivation. One potential limitation of this strategy is that the activation of certain cellular signaling pathways might require post-translational modification of their component molecules, such as phosphorylation or methylation, rather than mere overexpression. Thus, our screen system may not have revealed some of these genes and pathways, or those involved in repressing reactivation. The latter might be discovered by assays such as an siRNA library screen that specifically aims to inhibit endogenous gene expression.

To validate the biological relevance of our genome-wide screen, we performed detailed analysis of the signaling pathway from Ras to Ets-1. It is known that Ras-related pathways can be activated by a number of extracellular stimuli including growth factors vascular endothelial growth factor (VEGF) and platelet-derived growth factor (PDGF), as well as immunological cytokines and chemokines. Studies on KSHV have associated some of these stimuli with KSHV infection and Kaposi sarcoma pathogenesis. For example, it has been reported that VEGF-induced Raf activation promotes KSHV entry into cells [65] and that inflammatory cytokines can directly modulate KSHV replication [12,66]. Furthermore, it was shown that ERK1/2 and MEK1/2 induced by KSHV early during de novo infection of target cells are essential for expression of viral genes and for establishment of infection [67]. Our study directly revealed how Ras-related pathways play a role in herpesvirus reactivation. In both cases, the Raf/MEK/ERK pathway enhances the transcription of the RTA gene. It is biologically rational that the same cellular signaling pathways can induce viral reactivation as well as facilitate herpesvirus de novo infection.

In the in vitro culturing system, the cells are usually maintained in 10%–15% fetal bovine serum (FBS), where various growth factors and cytokines are present. Some of them are natural activators of certain cellular signaling pathways. Our study reveals an interesting phenomenon in that the intracellular processes can be triggered by further activation of these signaling pathways, and the induction level does not necessarily correlate with the different preexisting activation level of the related signaling components. KSHV-infected cell lines were shown to express a higher level of VEGF-A and B-Raf compared to uninfected cells, but the rate of spontaneous reactivation of latently infected cells is low, with variable levels of B-Raf in different cell lines [66,68]. This suggests that KSHV reactivation does not necessarily correlate with the endogenous Ras/Raf activation level, but is most likely balanced by other signaling pathways. We showed that additional activation of Ras could overcome a threshold to induce virus reactivation. Meanwhile, the preexisting Ras/Raf/MEK/ERK activity in the regular culturing condition could be an important factor that contributes to virus spontaneous reactivation, as indicated by a reduced spontaneous reactivation rate when this endogenous activity was inhibited by U0126. The spontaneous reactivation in a small percentage of cells represents cellular variation among “genetically identical cells.” There is no easy way to identify and isolate the cells that will permit virus reactivation before the reactivation actually takes place; thus, it is difficult to define the cellular variation that leads to spontaneous reactivation. It is intriguing that the Ras/Raf/MEK/ERK/Ets-1 signaling pathway identified via the genome-wide screen is critical for spontaneous reactivation as well. The role of this pathway in de novo infection, induced reactivation, and spontaneous reactivation implies that this pathway is an essential pathway selected by the virus to sense environmental cues at different stages of its life cycle.

The genome-wide screen approach utilized in this study provides an avenue by which the molecular signals regulating herpesvirus reactivation may be systematically identified and studied. The information revealed by the in vitro screen systems could greatly help identify potential physiological stimuli that can be further tested in in vivo studies. For example, the discovery of the role of PKA on KSHV reactivation led to the finding that physiological concentrations of epinephrine/norepinephrine effectively reactivate KSHV [37]. We also revealed that dopamine and dopamine derivatives can reactivate KSHV through dopamine receptors, which may function upstream of PKA and Ras (unpublished data). Thus, identification of the potential physiological stimuli, facilitated by the large scale screenings, provides a foundation for further verifications in in vivo systems.

The balance between latency and lytic replication is a fundamental virology question, particularly in the herpesvirus field. A better understanding of the mechanisms controlling the balance between latency and lytic replication may in turn enable the development of a more effective therapeutic strategy to treat KSHV and EBV-associated malignancies. For example, one potential approach to treat KSHV and EBV infection is to reactivate latent virus, followed by gancyclovir or acyclovir treatment to kill cells expressing lytic genes [69–73]. Based on the comprehensive knowledge of herpesvirus reactivation and the multiple signaling pathways that mediate this process, clinically applicable approaches to optimally reactivate herpesvirus in these malignancies could be developed, which in turn can facilitate the development of new therapeutic strategies.

## Materials and Methods

### Cell culture.

KS-1 cells are a gift from J. Said, and BC-3 cells are a gift from E. Cesarman. The construction of JSC-1 cells latently infected by rKSHV.219 reporter virus was described previously [53]. These B cells were cultured in RPMI 1640 medium containing 15% FBS. 293T cells were cultured in DMEM (Dulbecco's modified Eagle's medium) containing 10% FBS.

### Plasmids and reagents.

KSHV RTA cDNA was expressed in pFLAG-CMV2 vector [74]. Ha-Ras (Q61L) is a constitutively active form of Ha-Ras. Ha-Ras (S17N) is a dominant negative form of Ha-Ras. Raf22W and Raf22W/DDED are constitutively active forms of c-Raf [51]. The reporter construct pPAN-69Luc contains the PAN promoter region spanning nucleotides (nt) −69 to +14 in pGL3-basic vector [25], and the reporter pRpluc contains the 3-kb region upstream of the KSHV RTA coding sequence in pGL2-basic vector [23]. Reporter constructs pE4T and pE4T-RRE were generated as previously described [26]. The full-length ets-1 and truncated DN-ets-1 were cloned from cDNAs from BC-3 cells using primers 5′- CCC AAG CTT ATG AAG GCG GC −3′ (ets-1 forward primer), 5′- CCC AAG CTT ATG CCT GTC ATT C −3′ (DN-ets-1 forward primer) and 5′- GAA GAT CTT CAC TCG TCG GC −3′ (ets-1/DN-ets-1 reverse primer). Restriction enzyme sites Hind III and Bgl II were engineered into the 5′ and 3′ end of the sequence respectively, and both sequences were cloned into pFlag-CMV2 vector. Chemicals PD98059, U0126, SB203580, SP600125, and LY294002 were purchased from Calbiochem (http://www.emdbiosciences.com/html/CBC/home.html). The phospho-p38 MAPK (Thr180/Tyr182) (28B10) antibody, phospho-cJun (Ser63) antibody, and phospho-Akt (Ser473) antibody were purchased from Cell Signaling Technology (http://www.cellsignal.com).

### Establishment of the BC-3-G cell line.

The pPAN-122-d2EGFP construct was generated by replacing the promoter region in the pNFkB-d2EGFP vector (Clontech, http://www.clontech.com) with the PAN promoter region spanning nt −122 to +14 from the pGL3-8b construct [25]. pTW40 that contains the puromycin-resistant gene in pcDNA3 vector was generated previously in our lab. The BC-3-G cell line was established by cotransfecting pPAN-122-d2EGFP and pTW40 plasmids into BC-3 cells and selection with 1.5 ug/ml puromycin.

### High-throughput screen.

High-throughput (retro) transfections of the 26,000 mammalian cDNA library containing 15,000 full-length human clones (OriGene, http://www.origene.com), 4,000 additional full-length human cDNA clones (Mammalian Gene Collection; Open Biosystems, http://www.openbiosystems.com), and 7,000 full-length mouse cDNA clones (Mammalian Gene Collection, Open Biosystems) were carried out in a similar fashion as previously described [75]. In brief, 20 ng of reporter construct pPAN-69Luc, together with 62.5 ng of individual cDNAs, were incubated with 20 ul of serum-free medium containing FuGENE 6 (Roche, http://www.roche.com) in each well of 384-well plates. After 20 min of incubation, 10,000 KS-1 cells were delivered into each well. Luciferase activity was measured approximately 40 h post-transfection using Bright-Glo luciferase assay reagent (Promega, http://www.promega.com) on the Acquest multi-mode reader (Molecular Devices, http://www.moleculardevices.com). Control wells contained KS-1 cells cotransfected with pPAN-69Luc, and pcDNA6 vector treated with sodium butyrate and TPA.

### Reporter assays.

Transfections of 293T cells for Dual-Luciferase assays were conducted as follows: cells were seeded into 24-well plates 20 h prior to transfection so that they were ~90% confluent at the time of transfection. Then, 50 ng of the firefly reporter construct, 2 ng of Renilla luciferase construct pRL-SV40, and various amounts of cDNA expression constructs (supplemented with pcDNA3 vector DNA to a total DNA amount of 0.8–1 ug/well) were transfected using Lipofectamine 2000 (Invitrogen, http://www.invitrogen.com). Cells were incubated in DMEM containing 0.5% FBS for 24 h followed by Dual-Luciferase assays according to the manufacturer's instructions (Promega).

### RT-Q-PCR analysis.

KS-1/BC-3 cells were transfected by electroporation or using Lipofectamine 2000 (Invitrogen). For electroporation, 10^7^ cells were mixed with 9 ug of expression plasmid and 1 ug of pEGFP-C1 (BD Biosciences, http://www.bdbiosciences.com) as a transfection efficiency marker in a cuvette with a 0.4-cm gap (ISC BioExpress, http://www.bioexpress.com). Electroporation was performed using the Gene-Pulser II (Bio-Rad, http://www.bio-rad.com) with capacitance extender, 960 uF, 0.25 kV. At the indicated time points, cellular RNA was isolated using the RNeasy kit with on-column DNA digestion (Qiagen, http://www.qiagen.com). The mRNA was subsequently reverse transcribed into cDNA using SuperScript II RNase H-Reverse Transcriptase (Invitrogen). The primers used for RT-Q-PCR were: PAN primers (5′-GCCGCTTCTGGTTTTCATTG-3′ as the forward primer and 5′-TTGCCAAAAGCGACGCA-3′ as the reverse primer), viral thymidine kinase primers (5′-CGTAGCCGACGCGGATAA-3′ as the forward primer and 5′-TGCCTGTAGATTTCGGTCCAC-3′ as the reverse primer) and ORF65 primers (5′-GGCGGCCGTTTCCG-3′ as the forward primer and 5′-TCATTGTCGCCGGCG-3′ as the reverse primer). The PCR product amplified by GAPDH primers (5′-GAAGGTGAAGGTCGGAGTC-3′ as the forward primer and 5′-GAAGATGGTGATGGGATTTC-3′ as the reverse primer) was used as an internal control.

### EMSA.

Nuclear extract was prepared using a previously described method [76]. Ets-1 and Oct-1 DNA binding ability was assessed by EMSA using ^32^P-labeled double-strand oligonucleotides containing the Ets-1, mutated Ets-1, or Oct-1 consensus sequences with 5′ GGG overhang (Ets-1: 5′-GGGGTCAGTTAAGCAGGAAGTGACTAAC-3′, mutated Ets-1: 5′- GGGGTCAGTTAAGCAGGCAGTGACTAAC-3′, Oct-1: 5′-GGGAAGCTTTGTCGAATGCAAATCACTAGAACATATG-3′) [77,78]. Then, 2 μg of nuclear proteins were incubated with 2 × 10^4^ cpm of radiolabeled oligonucleotides in a binding buffer containing 1 μg poly (dI-dC), 0.25% NP-40, 5% glycerol, 10 mmol/L Tris HCl (pH 7.5), 50 mmol/L KCl, 1 mmol/L DTT, and 1 mmol/L EDTA for 20 min at room temperature. For competition experiments, excess amounts of cold Ets-1 or mutated Ets-1 oligo were added 10 min before the addition of labeled probe. Samples were separated on 4.5% acrylamide gels and assessed by autoradiography.

## Supporting Information

Figure S1 Effect of RTA on PAN Promoter Activity PAN-69Luc reporter and an increasing amount of RTA were transfected into 293T cells, and luciferase intensity was assessed.
(254 KB PDF)

Figure S2 Ras-Induced KSHV Lytic Protein Expression Assessed by Immunofluorescence Assay KS-1 cells were transfected with pcDNA3 or Ha-Ras (Q61L) by electroporation. Then, 72 h after transfection, cells were harvested, fixed, and subjected to immunofluorescence analysis. The total numbers of cells and cells expressing the lytic proteins v-IL-6, ORF59, and K8.1A were counted in three independent fields. v-IL-6 expression increased from 0.98% to 4.52%, ORF59 expression from 0.30% to 5.71%, and K8.1A expression from 0.20% to 1.80%. Transfection efficiency of KS-1 cells by electroporation ranges between 10% and 15%. One representative field for each lytic protein is shown. The total number of cells in each field is indicated by DAPI (4′,6-diamidino-2-phenylindole) staining. The percentage of cells that express the corresponding lytic proteins is shown below each pair of fluorescence pictures.
(686 KB PDF)

Figure S3 TPA-Induced KSHV Reactivation (A) BC-3 cells were pretreated with U0126 for 1 h before incubation with TPA (20 ng/ml) or sodium butyrate (1.5 uM) for 20 h, and K8 expression was assessed by Western blot analysis.
(B) BC-3 cells were cotransfected with MACS4.1 plasmid and DN-ets-1 24 h before incubation with TPA (20 ng/ml). Sixteen hours later, the successfully transfected cells were then enriched by a MACSelection system as +ve portion, and untransfected cells were indicated as −ve portion. K8 expression was assessed by Western blot analysis.
(348 KB PDF)

Figure S4 Effect of Specific Signaling Pathway Inhibitors The effect of SB203580 in the p38 pathway represented by an increased level of p-p38 (A), SP600125 in the JNK pathway represented by a reduced level of p-cJun (B), and LY294002 in the PI3K/Akt pathway represented by a reduced level of p-Akt (C) was shown by Western blots. BC-3 cells (A and C) or 3T3 cells (B) were serum starved overnight and pretreated with individual inhibitors for 1 h, followed by UV (400 uCi) and PDGF (50 ng/ml) treatment for 30 min. Protein lysates were prepared in RIPA buffer for Western blots to detect the phosphorylated proteins.
(347 KB PDF)

## Abbreviations

EBV: Epstein–Barr virus
EMSA: electrophoretic mobility shift assay
ERK: extracellular signal–regulated kinase
FBS: fetal bovine serum
GFP: green fluorescent protein
KSHV: Kaposi sarcoma–associated herpesvirus
MEK: mitogen-activated protein extracellular signal–regulated kinase kinase
ORF: open reading frame
PAN: polyadenylated nuclear RNA
PEL: primary effusion lymphoma
PKA: protein kinase A
RRE: replication and transcription activator response element
RTA: replication and transcription activator
RT-Q-PCR: reverse transcription–quantitative PCR
TPA: 12-O-tetradecanoylphorbol-13-acetate
v-Ki-ras2: Kirsten rat sarcoma 2 viral oncogene homolog
