# Supplementary figures and images for: Systematic Identification of Cellular Signals Reactivating Kaposi Sarcoma–Associated Herpesvirus
Source: PLoS Pathog. 2007 Mar 30;3(3):e44. doi: 10.1371/journal.ppat.0030044 (PMC1839163; doi:10.1371/journal.ppat.0030044)

Figure S1

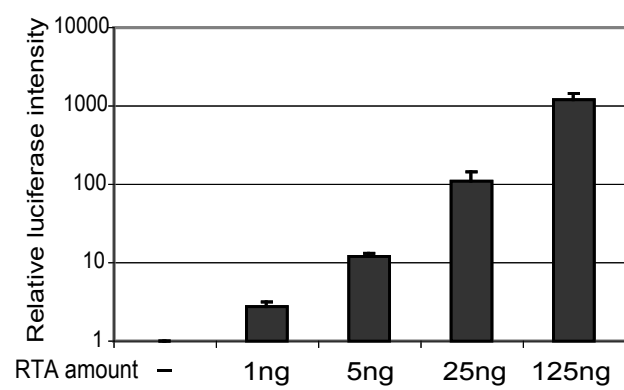

Supplement: Figure S1 — PAN-69Luc reporter and an increasing amount of RTA were transfected into 293T cells, and luciferase intensity was assessed. (254 KB PDF) [file ppat.0030044.sg001.pdf]

Figure S2

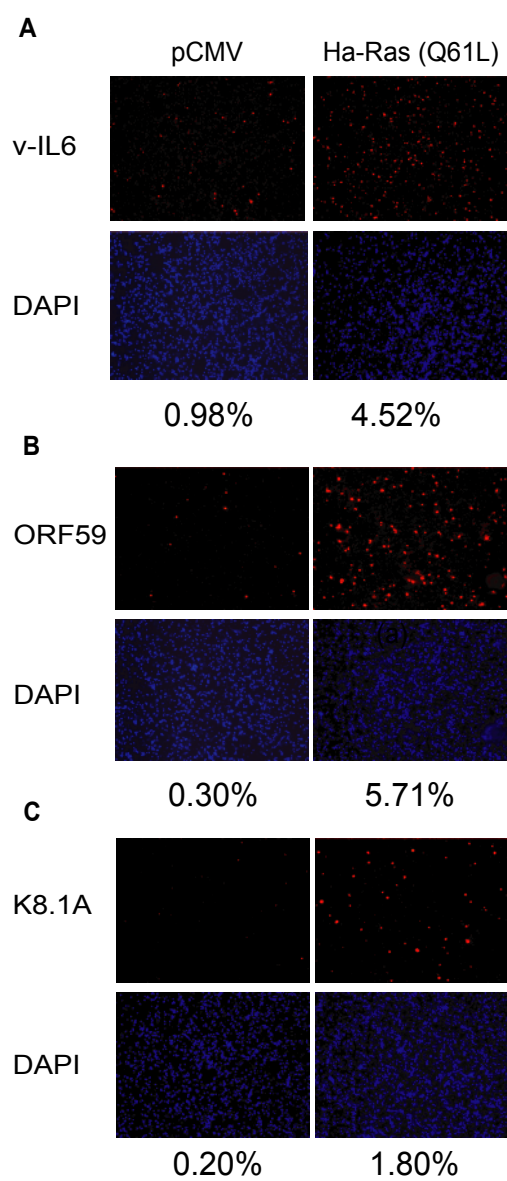

Supplement: Figure S2 — KS-1 cells were transfected with pcDNA3 or Ha-Ras (Q61L) by electroporation. Then, 72 h after transfection, cells were harvested, fixed, and subjected to immunofluorescence analysis. The total numbers of cells and cells expressing the lytic proteins v-IL-6, ORF59, and K8.1A were counted in three independent fields. v-IL-6 expression increased from 0.98% to 4.52%, ORF59 expression from 0.30% to 5.71%, and K8.1A expression from 0.20% to 1.80%. Transfection efficiency of KS-1 cells by electroporation ranges between 10% and 15%. One representative field for each lytic protein is shown. The total number of cells in each field is indicated by DAPI (4′,6-diamidino-2-phenylindole) staining. The percentage of cells that express the corresponding lytic proteins is shown below each pair of fluorescence pictures. (686 KB PDF) [file ppat.0030044.sg002.pdf]

Figure S3

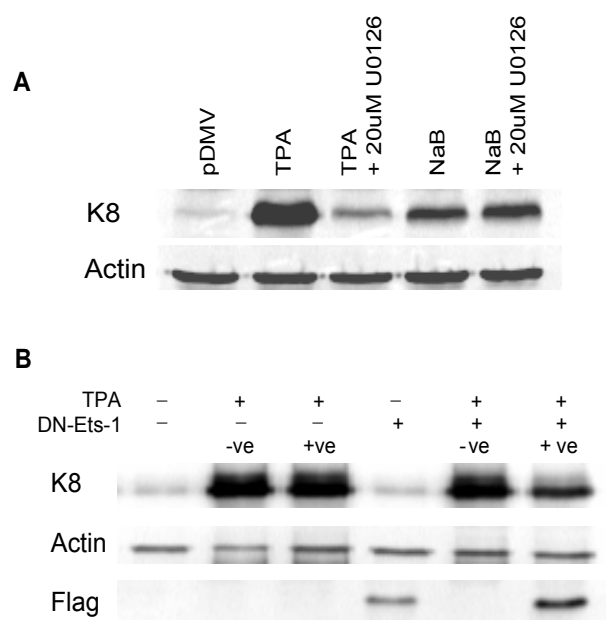

Supplement: Figure S3 — (A) BC-3 cells were pretreated with U0126 for 1 h before incubation with TPA (20 ng/ml) or sodium butyrate (1.5 uM) for 20 h, and K8 expression was assessed by Western blot analysis. (B) BC-3 cells were cotransfected with MACS4.1 plasmid and DN-ets-1 24 h before incubation with TPA (20 ng/ml). Sixteen hours later, the successfully transfected cells were then enriched by a MACSelection system as +ve portion, and untransfected cells were indicated as −ve portion. K8 expression was assessed by Western blot analysis. (348 KB PDF) [file ppat.0030044.sg003.pdf]

Figure S4

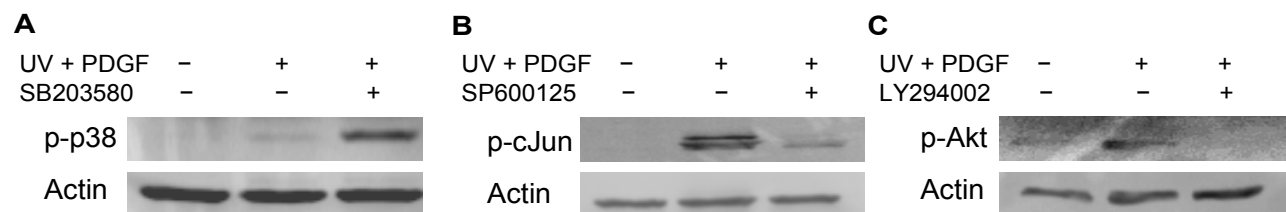

Supplement: Figure S4 — The effect of SB203580 in the p38 pathway represented by an increased level of p-p38 (A), SP600125 in the JNK pathway represented by a reduced level of p-cJun (B), and LY294002 in the PI3K/Akt pathway represented by a reduced level of p-Akt (C) was shown by Western blots. BC-3 cells (A and C) or 3T3 cells (B) were serum starved overnight and pretreated with individual inhibitors for 1 h, followed by UV (400 uCi) and PDGF (50 ng/ml) treatment for 30 min. Protein lysates were prepared in RIPA buffer for Western blots to detect the phosphorylated proteins. (347 KB PDF) [file ppat.0030044.sg004.pdf]
